# Validation Study on Iatrogenic Nerve Damage Reduction Using Augmented Reality on Elbow Phantom

**DOI:** 10.1016/j.mcpdig.2025.100221

**Published:** 2025-04-16

**Authors:** Giacomo Riberi, Antonio Cangelosi, Paolo Titolo, Elisa Dutto, Massimo Salvi, Filippo Molinari, Luca Ulrich, Marco Agus, Corrado Calì

**Affiliations:** a Department of Orthopedic and Traumatology II—Hand Surgery Unit, CTO Hospital, Città della Salute e della Scienza, Torino, Italy; b Department of Neuroscience “Rita Levi Montalcini,” Università degli Studi di Torino, Italy; c Intravides SRL, Torino, Italy; d DIGEP, Politecnico di Torino, Italy; e DET, Politecnico di Torino, Italy; f College of Science and Engineering, Hamad Bin Khalifa University, LAS Building, Doha, Qatar; g Neuroscience Institute Cavalieri Ottolenghi, Orbassano, Torino, Italy

## Abstract

**Objective:**

To compare augmented reality (AR) and classical intraoperative C-arm surgical navigation and evaluate whether head-mounted display improves surgical accuracy in the placement of a rod-like object, such as K-wire, using an anatomically accurate elbow phantom.

**Participants and Methods:**

Data were collected between January 10, 2024, and March 15, 2024. We developed an AR system, X-ray simulation system and surgical phantom to test K-wire placement in 3 locations of the distal humerus and proximal ulnar bones. An initial phase with only X-ray as guidance was performed as case control; in later phases, the candidates were allowed to also use the head-mounted display. The evaluation parameters were time, placement angle, number of X-ray images taken, number of attempts, and distance from anatomical structures.

**Results:**

In total, 19 physicians participated in the study. We analyzed 193 K-wire placements attempts that resulted in 150 estimated correct positions. This reflects a real-world scenario where multiple placements might be attempted to correctly place a K-wire. Compared with standard procedure, the use of AR resulted in −53.8 seconds in K-wire placement time, −47% of angular error from the K-wire target, −80% X-ray images taken to reach the estimate correct position, and decrease in distance variability of −81%, of the K-wire from anatomical structures of interest.

**Conclusions:**

Compared with C-arm, AR navigation improved time, and angle of placement, requiring less X-ray images.

Precision is one of the defining qualities of surgical practice, so much so that surgical precision is used commonly outside the medical context. In literature, it is commonly implied as a qualitative and quantitative evaluation parameter in studies correlating risk, damage, complications, and patient recovery.^1^ It can lead to improved determinacy, predictability, and controllability of surgical interventions reducing operative time and unwanted side effects for patients.^2^

In orthopedic surgery, iatrogenic peripheral nerve injuries constitute approximately 20% of traumatic nerve lesions, particularly prevalent following procedures to the upper and lower extremities.^3^^,^^4^ Preventive techniques are put in place to improve surgical performance and reduce nerve damage starting from training and knowledge of human anatomy. Nerve monitoring and high-resolution ultrasound has been described to evaluate intrasurgical status and regenerative potential of the major nerves of the upper limb.^5^^,^^6^ There are several indications that robotic-assisted surgery increases precision and reduces surgical sequelae.^7^ Minimally invasive surgery has instead been linked to an increased risk of iatrogenic damage following total hip arthroplasty.^8^

Augmented reality (AR) via head-mounted display has revolutionary potential for surgical education, preoperative planning, and intraoperative guidance in the surgical field,^9^, ^10^, ^11^ reducing placement error by half.^12^ This includes the use of commercial devices such as Microsoft HoloLens 2,^13^ custom solutions such as optical see-through head-mounted display that overlays C-arm imaging to the surgeon’s field of view,^14^ or simple visual instruction systems.^15^ Importantly, submillimeter precision has been achieved, making this approach suitable for orthopedic procedures in general.^16^, ^17^, ^18^

This study explores how using AR to visualize neurovascular anatomy during simulated minimally invasive orthopedic surgery could improve instrumental placement precision and reduce patient morbidity, possibly decreasing associated hospital costs. We previously developed an AR simulation system^19^ to enable precise K-wire insertion in a 3-dimensional (3D)-printed elbow phantom. This integration enables the visualization and precise placement of K-wires in predetermined locations, thereby ensuring a high level of accuracy and minimizing the risk of complications. This study therefore aimed to present an in-depth examination of the developed AR system and assess its efficacy in comparison with the conventional 2D visualization method offered by an X-ray C-arm.

In addition, we introduce several complementary approaches that further enhance procedural precision and practicality. These include a 3D atlas–based method for constructing custom anatomical phantoms, the integration of an affordable single point-of-view time-of-flight sensor for precise surgical navigation, and a simulated C-arm X-ray system. This multifaceted framework not only benchmarks AR against an established clinical tool but also offers practical, cost-effective solutions to advance surgical guidance research.

The results obtained in terms of risk reduction of injuries and improvement in surgical training are discussed, followed by the conclusions and future implications of this study for clinical applications. We describe a misplacement effect that affects AR navigation as well as similar navigation systems that should guide the application of this novel technology in surgical practice.

## Participants and Methods

### Materials

Our AR navigation system was evaluated on a surgical elbow phantom specifically designed for simulated procedures. Data were collected between January 10, 2024, and March 15, 2024. The virtual model can be manually placed in space by the user (Supplemental Video, available online at https://www.mcpdigitalhealth.org/), and the tracking system has an accuracy of 1.02 mm. Further technical details are provided in Supplemental Appendix (available online at https://www.mcpdigitalhealth.org/) and in the technical article by Cangelosi et al.^19^

### Elbow Phantom

The elbow phantom was designed to replicate key anatomical and surgical features essential for AR-guided training and validation. Anatomical data from the Z-Anatomy Atlas^20^ (adapted for molding and 3D printing via Autodesk Fusion 360). We selected 3 target K-wires for candidates to aim at Bones were fabricated using polylactic acid 3D printing with a gyroid infill pattern (20%) to not only mimic cortical and trabecular bone properties but also enable ease of perforation. Silicone-based soft tissue was molded using room temperature vulcanizing silicone with Shore A5 hardness (Supplemental Figure 1, available online at https://www.mcpdigitalhealth.org/).

The phantom included 10 surface markers, 5 on the top and 2 on the bottom, to facilitate precise alignment between the physical model and its virtual counterpart. A hollow cavity within the polylactic acid bones further enhanced realism by emulating natural bone material diversity.

The hardware setup included a GSB 18V-55 Bosch drill fitted with reflective markers for tracking ensuring accurate K-wire placement. A rigid stand stabilized the phantom (Supplemental Figure 2, available online at https://www.mcpdigitalhealth.org/), replicating its virtual alignment.

### Software Packages

Three-dimensional image capture and analysis are performed by the ComputerVisionModule software that retrieves data from the Kinect Azure DK camera and computes 3D positions for each of the 8 markers, 4 from the phantom and 4 from the drill, which are virtually categorized as being part of the former or the latter, based on 3D shape. The Kinect’s infrared depth camera enabled robust marker detection under various lighting conditions. By contrast, an earlier attempt using visible-spectrum QR code tracking proved unreliable owing to lighting variability and operator dependency.^21^ The position and orientation of the phantom and surgical drill is then used by the X-ray simulator and AR navigation system software to render a 3D real-time hologram of the tool, K-wire target, and critical anatomical structures on the Microsoft HoloLens 2. This integration allowed surgeons to visualize the surgical environment dynamically and adjust their approach accordingly. The software also displays real-time, 2D gray-scale X-ray images on a secondary monitor, effectively simulating an X-ray C-arm (Supplemental Figure 2). Custom-designed software packages for data collection are described in Supplemental Appendix and are available on GitHub.

### Protocol

Participants in this study performed 3 phases:

- Phase 0 (control): 3 K-wire target insertions using only standard 2D X-ray visualization.
- Phase 1: 3 K-wire target insertions using AR and 2D X-ray for guidance.
- Phase 2: 3 K-wire target insertions using AR and 2D X-ray for guidance to evaluate the learning curve.

For each K-wire target, candidates performed multiple placement attempts (PAs) until they are satisfied, declaring the PA as successful and reaching the estimated correct placement (ECP). After reaching the ECP for a target, the candidate is allowed to move on to the next target or end the phase after all 3 targets have reached ECP.

### Data Collection

At the beginning of the test, each candidate was asked about demographic information, experience, and eyesight condition. For each PA, time durations, radiation exposure, 3D positioning, and anatomical data are recorded on Autodesk Fusion 360 and a local SQLite3 database. In addition, data included the result of the placement (successful or failed) and candidate subjective information such as confidence about the angle and position. Statistical data such as positioning attempt counts and positioning attempt counts failed relative to each ECP and phase were recorded for each candidate.

### Statistical Analyses

Phase, ECP, and PA database tables were anonymized, and we computed statistical, positional, duration, fluoroscopic image count, and anatomical analyses.

## Results

A more detailed narrative of the results is present in Supplemental Appendix. In this study, 19 applicants performed 150 ECPs for a total of 193 PA across 50 phases. A total of 11.8 hours were spent for surgical simulation, together with 2542 fluoroscopic images. A rendering of all PAs can be seen in Supplemental Figure 3 (available online at https://www.mcpdigitalhealth.org/); 34 (18.1%) PAs hit arteries, veins, or nerves with 43 contact instances, and 31 (21.3%) ECPs hit arteries, veins, or nerves with 43 contact instances.

### Participant Characteristics

Eight medical master’s students, 10 orthopedic residents, and 1 orthopedic surgeon participated in the study (Supplemental Table 1, available online at https://www.mcpdigitalhealth.org/). The reported operation experience (*P*<.01) and age (*P<.*01) were linked with the participants’ experience level, as expected (Supplemental Table 2). The participants’ sex, familiarity with virtual reality/AR technology, and proficiency in 3D software visualization did not conform to this pattern.

### Statistics

**Figure 1:**
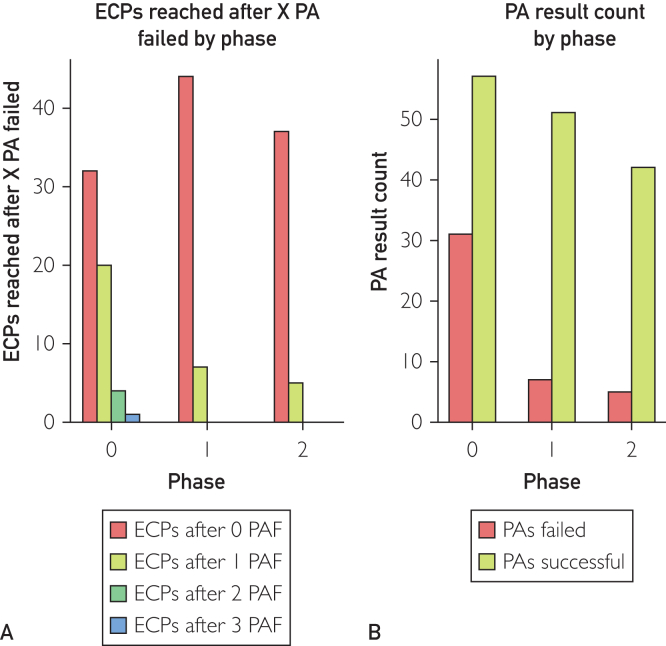
(A) The count of estimated correct placements (ECPs) that were reached after 0, 1, 2, and 3 failed placement attempts (PAs) with phase as predictor variable. ECPs after 0 positioning attempt counts failed (PAF) for phase 0, 1, and 2 is 56.1% (n=57), 86.3% (n=51), and 88.1% (n=42; total, N=150), respectively. The χ^2^ between phase 0, 1, and 2 is 20.85 with a *P*<.005. (B) PA success rate for each phase. Success rate for phase 0, 1, and 2 is 64.8% (n=88), 87.9% (n=58), and 89.4% (n=47; total, N=193), respectively. The χ^2^ between phase 0, 1, and 2 is 15.69 with *P*<.005.

The number of ECPs reached after 1, 2, or 3 failed PA displayed a statistically significant drop for phase 1 and 2 where AR was used (Figure 1A). Success rate increased by 23.1% between phases 0 and 1 and 24.6% between phases 0 and 2 with a *P*<.005, proving a statistically significant improvement (Figure 1B).

### Duration

**Figure 2:**
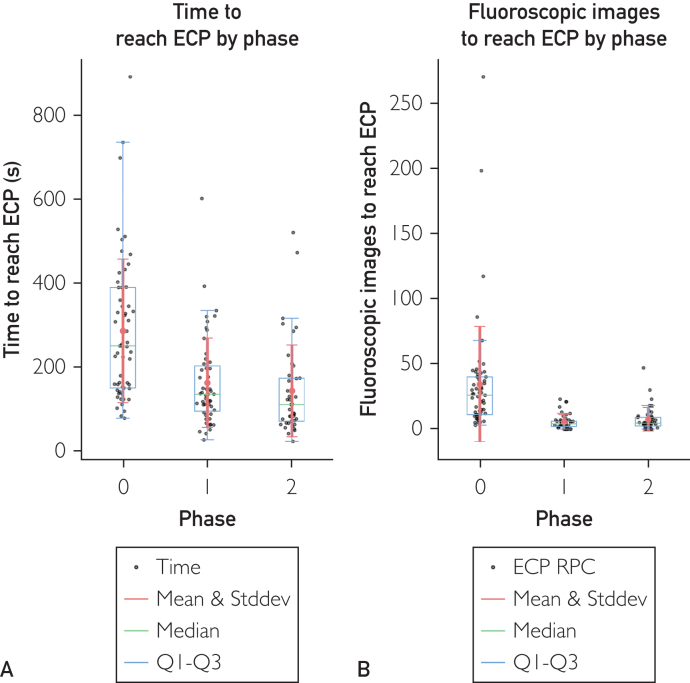
(A) Duration of estimated correct placements (ECPs), with phase as predictor variable. The mean time duration for phase 0, 1, and 2 is 285.76 seconds (n=57), 162.29 seconds (n=51; −123.47 seconds or −43% compared with phase 0), and 142.81 seconds (n=42; −142.96 seconds or −50% compared with phase 0; total, n=150). The ANOVA test *f* value between the 3 phases data is 17.10 with a *P*<.005. The Dunnett test stat-value of phases 1 and 2 with phase 0 as control group is −4.73 (*P*<.005) and −5.19 (*P*<.005), respectively. The 95% CI is −181.92 to −65.02 seconds and −204.62 to −81.29 seconds, respectively. (B) The X-ray images acquired to reach each ECP with phase as predictor variable. The mean number of images taken for phase 0, 1, and 2 is 34.30 (n=57), 5.43 (n=51; −28.87 or −84% compared with phase 0), 7.38 (n=42; −26.92 or −78% compared with phase 0; total, n=150), respectively. The ANOVA test *f* value between the 3 phases data is 17.86 with a *P*<.005. The Dunnett test stat-value of phases 1 and 2 with phase 0 as control group is −5.37 (*P*<.005) and −4.75 (*P*<.005), respectively. The 95% CI is −40.90 to −16.83 and −39.62 to −14.22, respectively.

A clear and statistically significant time reduction is visible in Figure 2A, and Supplemental Tables 3 and 5, which is −123.47 seconds between phases 0 and 1 and −142.96 seconds between phases 0 and 2 (*P*<.005, ANOVA and Dunnett test).

### Fluoroscopy

The mean reduction of fluoroscopic images required to reach ECP (Figure 2B, and Supplemental Tables 3 and 5) was −28.87 (−84%) between phases 0 and 1 and −26.92 (−78%) between phases 0 and 2. (*P*<.005, ANOVA and Dunnett tests).

### Position

**Figure 3:**
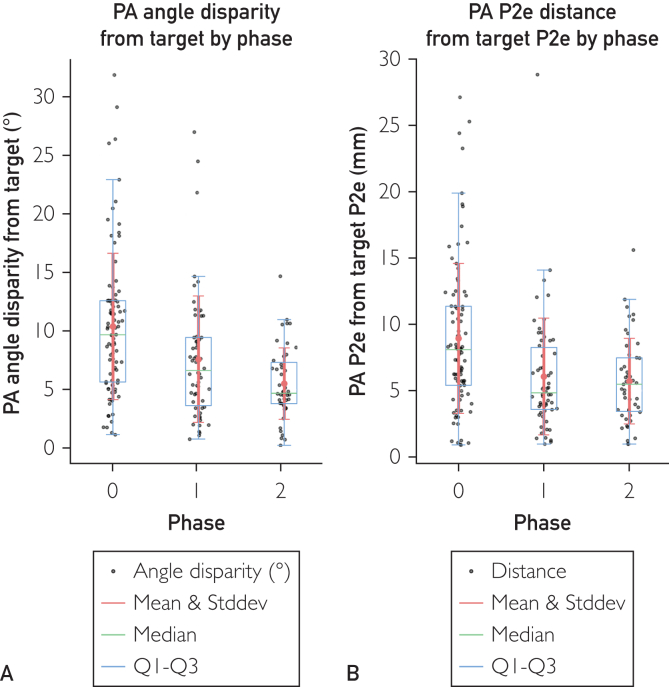
(A) The Δangle of each placement attempt (PA) axis from the target axis with phase as predictor variable. The mean angle for phase 0, 1, and 2 is 10.40° (n=88), 7.60° (n=58; −2.80° or −27% compared with phase 0), and 5.52° (n=47; −4.88° or −47% compared with phase 0; total, n=193), respectively. The SD for phases 0, 1, and 2 is 6.24°, 5.38° (−0.86° or −14% compared with phase 0), and 3.05° (−3.19° or −51% compared with phase 0), respectively. The ANOVA test *f* value between the 3 phases data is 13.54 with a *P*<.005. The Dunnett test stat-value of phases 1 and 2 with phase 0 as control group is −3.09 (*P*<.005) and −5.04 (*P*<.005), respectively. The 95% CI is −4.83° to −0.77° and −7.05° to −2.71°, respectively. (B) The distance between skin insertion point of PA K-wire and skin insertion point of target K-wire with phase as predictor variable. The mean distance for phase 0, 1, and 2 is 8.92 mm (n=88), 6.04 mm (n=58; −2.88 mm or −32% compared with phase 0), and 5.69 mm (n=47; −3.22 mm or −36% compared with phase 0; total, n=193), respectively. The SD for phase 0, 1, and 2 is 5.64, 4.40 (−1.23 mm or −22% compared with phase 0), and 3.22 mm (−2.42 mm or −43% compared with phase 0), respectively. The ANOVA test *f* value between the 3 phases data is 9.69 with a *P*<.005. The Dunnett test stat-value of phases 1 and 2 with phase 0 as control group is −3.56 (*P*<.005) and −3.73 (*P*<.005), respectively. The 95% CI is −4.69 to −1.06 mm and −5.16 to −1.29 mm, respectively.

The mean angle error dropped in a statistically significant manner: compared with phase 0 (X-ray) by −2.80° (−27%) for phase 1 (AR first try) and −4.88° (−47%) for phase 2 (AR second try), indicating a substantial improvement in PA angular precision with AR (Figure 3A, and Supplemental Tables 3 and 5). This reduction is statistically significant (*P*<.005, Dunnett test between phases 0 and 1, *P*<.001 between phases 0 and 2).

In addition, mean angle and SD reduced significantly, indicating an increase in PA angle consistency (*P*<.005 between phases 0 and 2 and *P*<.05 between phases 1 and 2, Levene test with Bonferroni correction). No significant change was found between phases 0 and 1.

The mean PA entrance point distance from target also reduced significantly (Figure 3B, and Supplemental Tables 3 and 5): compared with phase 0 (X-ray) by −2.88 mm (−32%) for phase 1 (AR first try) and −3.22 mm (−36%) for phase 2 (AR second try), indicating a substantial improvement in PA precision. The SD displayed a progressive reduction, indicating a significant reduction in variability, and thus an increase in consistency and precision, of the PA skin insertion point distance from target skin insertion point (Figure 3, and Supplemental Tables 3 and 5).

### Anatomy

**Figure 4:**
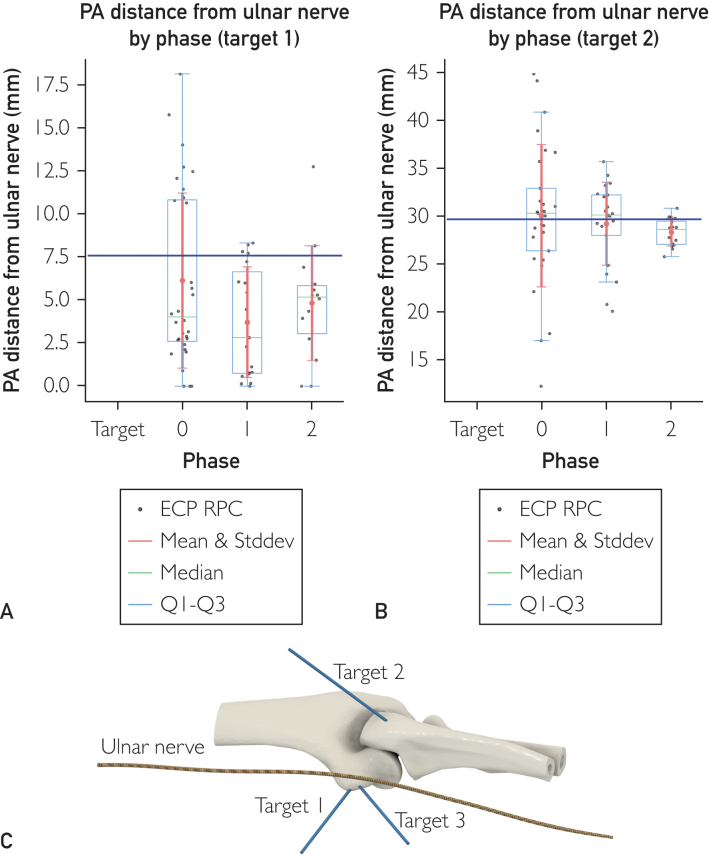
(A) The distance between placement attempt (PA) K-wire aiming at K-wire target 1 and ulnar nerve with phase as predictor variable. The target distance corresponds to 7.56 mm. The mean PA distance for phases 0, 1, and 2 is 6.12 mm (n=32; −1.44 mm or −19% compared with target), 3.71 mm (n=19; −3.85 mm or −51% compared with target), and 4.81 mm (n=14; −2.75 mm or −36% compared with target; total, n=65), respectively. The SD for phases 0, 1, and 2 is 5.07 mm, 3.20 mm (−1.87 mm or −37% compared with phase 0), and 3.31 mm (−1.76 mm or −35% compared with phase 0), respectively, indicating a reduction in the variability of PA distance from ulnar nerve leading to an increase of consistency. The ANOVA test *f* value between the 3 phases data is 1.41 with a *P*=.25. The Dunnett test stat-value of phases 0, 1, and 2 with target as control group is 0 (*P*=1.00), −1.83 (*P*=.18), and −0.90 (*P*=.72), respectively. The 95% CI is −2.73 to 2.73 mm, −5.58 to 0.75 mm, and −4.81 to 2.19 mm, respectively. The Levene test for the SD exhibits nonsignificance for all phase combinations: 0-1-2, 0-1, 0-2, and 1-2. (B) The distance between PA K-wire aiming at K-wire target 2 and ulnar nerve with phase as predictor variable. The target distance corresponds to 29.62 mm. The mean PA distance for phase 0, 1, and 2 is 30.00 mm (n=29; +0.38 mm or +1% compared with target), 29.19 mm (n=21; −0.43 mm or −1% compared with target), 28.30 mm (n=16; −1.32 mm or −4% compared with target; total, n=66), respectively. The SD for phases 0, 1, and 2 is 7.41 mm, 4.32 mm (−3.09 mm or −42% compared with phase 0), and 1.45 mm (−5.69 mm or −80% compared with phase 0), respectively. The ANOVA test *f* value between the 3 phases data is 0.33 with a *P*=.80. The Dunnett test stat-value of phases 0, 1, and 2 with target as control group is 0 (*P*=1.00), −0.46 (*P*=.94), and −0.88 (*P*=.72), respectively. The 95% CI is −3.90 to 3.90 mm, −5.07 to 3.44 mm, and −6.33 to 2.92 mm, respectively. The Levene test for the SD exhibits significance for the phase combinations: 0-2 (9.44; *P*<.01 after Bonferroni correction) and 1-2 (6.33; *P*<.05 after Bonferroni correction). (C) rendering of the position of the elbow joint, the 3 target positions, and the ulnar nerve, as shown in superimposition to the surgical phantom.

For target 1 on the phantom, PAs resulted in a nonsignificant change in the mean distance from the ulnar nerve across phases 0, 1, and 2 (Figure 4A). The mean PA distance from ulnar nerve from target 2 remained relatively stable across phases 0, 1, and 2 (Figure 4B). The SD steadily decreased across the 3 phases, indicating a reduction in the variability of PA distance from ulnar nerve, leading to an increase of consistency (Supplemental Table 4). We observed a statistically significant variance reduction, which correlates with an improvement in placement precision. Specifically, the variance difference between phases 1 and 2 was 6.33 (*P*<.05, Levene test, Bonferroni corrected), and the difference between phases 0 and 2 was even greater, at 9.44 (*P*<.01, Levene test, Bonferroni corrected).

## Discussion

The primary objective of this study was to compare the performance of physicians using 2 visualization systems (AR visualization vs standard X-ray C-arm). In our setup, a monitor displays the data in 2D, whereas the HoloLens renders the same data in 3D (Supplemental Figure 1 and Supplemental Video). Computing setup is identical in both cases, ensuring that any significant precision errors that might affect outcomes in a real-world scenario,^22^ are not a confounding factor in our comparative analysis. Technical aspects of our current setup have been analyzed elsewhere.^19^

The AR model can be placed freely in space instead of being directly overlaid on the surgical phantom. Although an automatic overlay might seem intuitive, feedback from surgeons revealed that AR superimposition works well only in a quasistatic surgical field viewed through a semitransparent display. Positioning the model adjacent to the surgical site offers several advantages: it reduces visual clutter, avoids occluding critical anatomical landmarks, and permits a clear view of the entry point while still benefiting from AR guidance.^23^

### Study Results

AR features a statistically significant improved ratio of PA successful over PAs failed (Figure 1B). Furthermore, the number of retakes to reach the ECP decreased significantly across the 3 phases (Figure 1A). We observed an improvement of the ratio from 64% PA successful in phase 0 to 89% in phase 2. This obviously impacts the time needed to reach ECP shortening operation time and reduces the potential damage to the patient.

Time duration of execution was also characterized by a 50% reduction from phase 0 to phase 2, with a clear curve of improvement (Figure 2A). Looking at the learning curve, we suspect that it might be reduced even more with more learning time available. More studies and data gathering are needed to further analyze the learning curve effect on time to reach ECP.

The analysis of fluoroscopic images used to reach ECP was also in accordance with the first 2 presented with a −78% reduction (Figure 2B). This impacts the surgery time, the machine wear and the risks associated with the radiation exposure for both the patient and surgeons.

The 2 positional parameters showed a statistically significant improvement, with the PA angle disparity from the target reduced by 47% (Figure 3A) and the PA skin entrance point distance from the target (Figure 3B) reduced by 36%. These reductions indicate meaningful improvements in K-wire placement precision when using AR compared with that using only X-ray.

Aside from simple parameters such as statistics, duration, fluoroscopic images, and positional, the most surgically relevant are the anatomical considerations. It is interesting to note how in Figure 4 the decrease in SD is noticeable and statistically significant for target 2 (Figure 4B) but not for target 1 (Figure 4A).

First, we must differentiate the 2 cases with different relative positions of the target in respect to the ulnar nerve in the 3D rendering of Figure 4C. Target 1 is positioned dangerously close to the ulnar nerve, whereas target 2 is located at a safe distance. Furthermore, target 1 closest point to the ulnar nerve is quite superficial under the skin and the opposite is true for target 2. Although targeting the K-wire target 1, some K-wire PA hit the ulnar nerve both using only X-ray or X-ray/AR combo. We can explain this effect as follows. For the placement of a rod-like object such as a screw, screwdriver, drill bit, or as in our case a K-wire, we must consider errors in both the insertion point and the angle of positioning. Combined, these errors can be visualized as a misplacement cone, specifically a conical frustum. The 2 parameters defining the conical frustums of phases 0, 1, and 2 can be found in Figure 3.

We analyzed the K-wire passage probability in space layer by layer at different depths (z axis) and suppose that the probability of a K-wire passing in a specific point of the layer plane is described by a gaussian cone centered on the target axis. This means that the probability of hitting a 2D structure at that level can be computed as the volume under the curve inscribed by the structure surface (a nerve in our case). The Gaussian parameters sigma X and sigma Y, which characterize the curve, are calculated to ensure that the circumference of the conical frustum at various depths, encloses 50% of the volume beneath the curve. We can therefore postulate that a rod-like object placed by insertion targeting a rod-like position has an intrinsic positioning error, which can be visualized as a conical frustum and more correctly described as Gaussianoid conical frustum (GCF).

**Figure 5:**
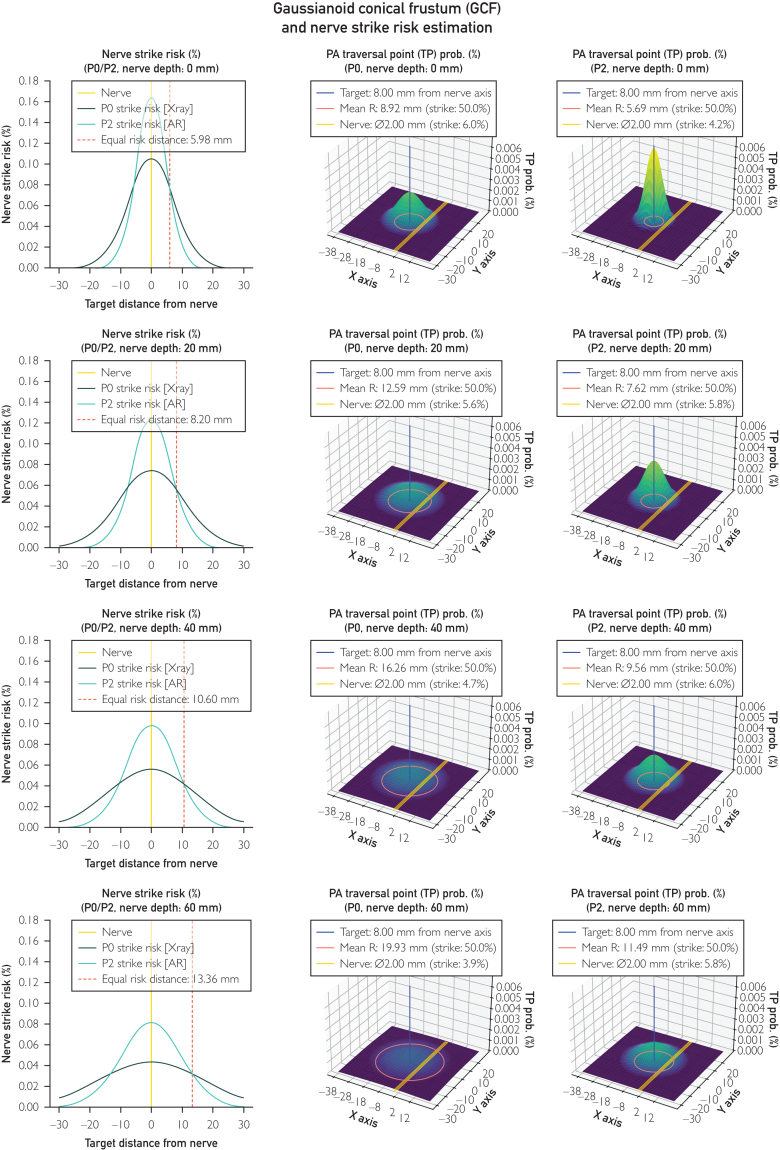
Graphical render of the Gaussianoid conical frustum (GCF) over 4 progressively deeper planar sections: (A), 0.0 mm; (B) 20.0 mm; (C) 40.0 mm; and (D) 60.0 mm, on the second and third columns. Nerve strike risk estimation, the representation of the effect, which describes a paradoxical increase of risk for targets within equal risk distance (ERD), is displayed in the first column.

Visualization and statistical analysis of the GCFs from phase 0 (X-ray only) and phase 2 (X-ray and AR) are rendered in Figure 5. The second and third columns render 4 planar sections of the 2 GCFs with a nerve drawn in yellow, the target in blue and the mean distance from the target as an orange circle. The conical frustum can be seen across the 4 sections as an enlarging orange circle. The 3D gaussian curve represents the probability that a K-wire, targeting the blue axis, will pass through the plane section in each position. Although the second and third columns help visualize the GCF, the first column shows the probability that a K-wire placed in phases 0 and 2 will hit the nerve as a function of the target’s distance from the nerve.

The equal risk distance (ERD) is highlighted with a red dot: K-wires aiming at a target within this distance from the nerve have a greater risk of striking it. This effect is observable when comparing the misplacement probability described by 2 GCFs, which make the relative risk dependent on not only the navigation method used but also the target distance.

The effect resulting from the GCFs seems paradoxical because we would expect AR to decrease the risk of damaging nearby structures by enhancing precision (Supplemental Figure 4). This is true only for distances greater than the ERD. However, although AR increases the damage risk to nearby structures when the target is within the ERD, it is important to consider other factors that justify its use and the use of similar navigation technologies. First, our goal is not only to avoid hitting nerves but also to accurately place a K-wire in a specific, predefined location. Second, there are numerous other sensitive tissues in the surrounding area, and depending on the surgical site, these will increase the likelihood of iatrogenic tissue damage in a real surgical scenario. Obviously, this concept does not apply only to AR and K-wires but also to any other similar axial navigation systems. This consideration is a good reminder that, even with a tool that augments precision, the surgeon should always prefer the safest spot and/or trajectory when planning surgery.

## Conclusion

This study tested a cost-effective AR system to simulate minimally invasive orthopedic surgery. We integrated a separate X-ray simulator that integrates 3D navigation infrared markers. Originally designed as a control method for this study, it also holds potential for extensive training in adapting to 2D visualization, an essential skill in many surgical procedures.

Our results suggest that AR could become a valuable aid in surgery, benefiting both surgeons and patients. However, larger participant groups are needed to strengthen ours and other studies observations. Beyond scientific validation, future development should focus on refining the system for real operating room applications, moving from a 3D model simulation to real surgical settings. With continued advancement, AR could not only transform surgical navigation but also serve as a powerful training tool, helping residents and new surgeons gain critical skills and potentially lowering patient complication risks.

## Potential Competing Interests

Cangelosi reports departmental resources from Università degli Studi di Torino and Politecnico di Torino, Torino. Dr Cali reports grants from University of Turin and is the founder and president of the academic startup Intravides. All other authors report no competing interests.

## Ethics Statement

IRB approval was not obtained because this study did not involve patients.
