# Supplementary material for: Validation Study on Iatrogenic Nerve Damage Reduction Using Augmented Reality on Elbow Phantom
Source: Mayo Clin Proc Digit Health. 2025 Apr 16;3(2):100221. doi: 10.1016/j.mcpdig.2025.100221 (PMC12141047; doi:10.1016/j.mcpdig.2025.100221)
Supplement: Supplementary Material [file mmc1.pdf]

Validation study on iatrogenic nerve damage reduction using Augmented Reality on elbow phantom: supplementary

Methods

Protocol

Experiment execution workflow is visible in the vid. S4. Note that only a PA is done for each of the three targets, meaning that each PA was declared successful and ECP was reached at first try. It's important to keep in mind that between each PA a manual or automated measurement of the 3D position of the K-wire was recorded on the database and 3D modeling software.

Phantom design and construction

The workflow developed to design the organ model from medical imaging data is shown in fig. S1. The human elbow model was extracted from Z-Anatomy Atlas (fig. S1.1 and S1.2) and adapted for molding and printing on Autodesk Fusion 360 (fig. S1.4). Together with an expert orthopedic surgeon 3 target K-wires were placed on surgically accurate positions in the virtual model (fig. S1.3). The bones are then fixed to the mold A and B with screws via alignment rails to improve reproducibility (fig. S1.5). Base Silicone is mixed with the catalyst with a 100:5 ratio as indicated by the producer and a 0·5% by weight of E124 red food coloring powder is added to enhance the simulation model's skin-like pink appearance (not shown in fig. S1). The mix is rapidly poured in the assembled mold (fig. S1.6) and for 20 minutes the assembly is agitated to reduce silicone bubbles formation. Curing process takes 12 to 24 hours at room temperature after which the phantom can be extracted (fig. S1.7).
The structures that are not bone, such as the skin, muscles, tendons, arteries, veins, and nerves, are designed to be soft tissue. All 3D information pertaining to the internal anatomical structures is only included in the virtual model.
RTV silicone (silicone R5, Reschimica s.r.l., Arezzo, Italy) with hardness shore A5 was used for the molding of soft tissue as it allows rapid production and enables the customization of case-specific phantoms tailored to distinct anatomical geometries. This material stands out as a cost-effective option with minimal shrinkage, by default presenting a similar consistency to human tissue but allowing it to be further customized to mimic various tissue properties, rendering it an exemplary material for fabricating high-fidelity surgical training phantoms.[1,2] The RTV silicone phantom surface features 10 markers, with 5 positioned on the top and 5 on the bottom around the arm's circumference. These markers serve as reference points with known 3D locations, both virtually and on the physical model, to facilitate accurate spatial alignment.
The phantom bones are constructed using 3D printed PLA (PolyLactic Acid) exploiting attributes of sub-millimeter precision and automation allowing for near-perfect matching between virtual and physical models. This method has already been documented in the literature for its high degree of part customization, accurate replication of several human body parts, and ability to modify the density of certain portions by adjusting the proportion of infill in the 3D printed part.[3] The bones have a central hollow cavity to imitate the structure of cortical and trabecular bone.

The infill percentage of the prints is gyroid 20% to reduce density, ease of perforation and reduction perforation induced plastic burr.[4]

Hardware

In a simulated surgical environment (fig.S2), a surgical field limited to essential elements proves both practical and effective.[5] Following Aumann's approach, this reduction retains only the core functionalities crucial for the procedure, enabling a focused evaluation of AR in surgical training without the complexities and expenses of a full-scale operating room.[6] This streamlined approach supports effective training while preserving a controlled, repeatable simulation setting.[7,8]

The surgical testing setup is organized to reproduce a standard surgical environment and navigator configuration for K-wire placement: the surgeon, carrying the drill (fig.S2.2), is in front of the patient (fig.S2.1) with the X-ray screen (fig.S2.4) placed in a comfortable relative position. The infrared camera (fig.S2.3) is set laterally on a tripod in a way that can have constant line of sight with the infrared (IR) spheres of the phantom and drill (well visible in fig.S2.2 and S2.6).

This framework supports simulated C-arm views (fig. S2.4) and offers real-time surgical AR guidance (fig. S2.6) using Microsoft's HoloLens 2 (fig.S2.5). Simulated preoperative surgical planning (fig.4, below) was used to identify critical anatomy for intervention. By incorporating K-wire targets into the virtual anatomical model, the HoloLens 2 can superimpose real time location of the K-wire on the AR phantom.

The phantom is positioned on a stand consisting of pillars, adapters, and interconnecting elements for the stability and proper positioning (fig.S2.1). It is a rigid structure closely reproducing the virtual design so that IR spheres can be attached to the PLA plastic instead of the phantom silicone.

The GSB 18V-55 Bosch drill (fig.S2.2) is equipped with a tracking adapter which is mounted with reflective IR spheres allowing for clear visibility and tracking in space by the 3D camera (fig.S2.3).

Kinect Azure DK depth camera captures the surgical field containing the 8 IR spheres to track relative positions of phantom and surgical drill (fig.S2.6). It is set in Wide field-of-view (WFOV) mode, meaning the capture sensor is 1024×1024 pixels wide and running at 5fps. Minimum and maximum sensor distance are respectively 0·25m and 2·88m. In our previous setup, we aimed to eliminate the need for an external camera by relying solely on the onboard camera of the HoloLens 2, utilizing VuForia QR code tracking in the visible light spectrum. However, this approach proved to be unreliable, with performance heavily influenced by external lighting conditions and the operator's experience.[9]

The Microsoft HoloLens 2 AR visor is used as a semi-transparent 3D display to present surgical phantom models, along with the real-time positioning data of a K-wire tool relative to pre-defined surgical targets. This HMD employs both IR and visible light cameras to capture detailed spatial information (blue arrows). The IR cameras are particularly useful for depth sensing and can function effectively in low-light conditions, enhancing the device's ability to understand its surroundings. This implies that it can accurately detect the wearer's position inside the space, allowing the virtual model to be fixed within the real environment.

K-wires are made from hardened stainless steel, each 108 mm long and 1·6 mm in diameter reproducing the ones used currently in the surgical field. A diamond tip type was chosen to improve performance during insertion, to reduce PLA plastic burr and for ease of construction.

Software

Tracking System

NIOTS are widely adopted in clinical settings due to the limited ambient light interference.
To reproduce their framework, Kinect Azure DK was chosen for its infrared Time-of-Flight depth camera. Reflective markers were segmented from IR images using an intensity threshold and CHT. Subsequently, 3D point cloud data was processed to compute the centroids of the segmented markers.
The correspondence problem was solved using Procusean Analysis. The resulting 3D coordinates of the target frame were sent via UDP to X-Ray simulation software and AR guidance system for 2D and 3D rendering respectively.
The open-source HoloElbow-ComputerVisionModule framework is accessible on GitHub at

- https://github.com/Antocg99/01-HoloElbow-AR
- https://github.com/Antocg99/01-HoloElbow-ComputerVisionModule

X-Ray Simulator and AR Navigation System

The X-ray emulator simulates fluoroscopic imaging of a patient's elbow. Models were extracted from the z-Anatomy atlas and refined with Autodesk Fusion 360. The simulated C-Arm interface mimics essential features of a real fluoroscope and additionally provides data such as X-ray exposure count, tool tracking status and angulation. These parameters enhance understanding of the simulation context. For enhanced simulation accuracy, a photograph can be captured by pressing a foot pedal located beneath the phantom, while left and right pedals allow for control of the C-arm's rotational position. Data collection is facilitated by the "kwire-virtualization-system" software that allows virtualization of PAs into Autodesk Fusion 360 and advanced 3D computations such as 3D distances and angles. Data storage is performed by the "AR-kwire-placement-test-companion" software that logged to a SQLite3 database surgical parameters, tool trajectories, and operator performance metrics for post-test analysis.
All of the aforementioned software packages were custom-designed for the experiment, leveraging publicly available software libraries. The source code for these four systems is accessible on GitHub.

The AR Guide System runs in HoloLens 2 through the Holographic Remoting tool. It overlays the anatomical digital model onto the simulated surgical field. Rather than automatically overlaying the virtual model onto the real phantom, our system allows for manual alignment, a process whose accuracy depends on the user's skill and the precision of eye calibration. Registration is achieved by aligning the real and virtual models within a tracking-defined virtual space, with the origin established at the optical center of the Kinect's IR camera. Although multiple accuracy metrics exist, point-to-point accuracy is most relevant in this context. Critical nerve and vessel locations are visible in the Field of View, aiding navigation and decision-making to reduce potential risks. Users can adjust the digital anatomy for ideal positioning and examine surgical approaches from various angles. Measurements between corresponding points in the real and virtual spaces revealed an average accuracy of 1.02 mm, with a standard error of 1.67 mm across 42 measurements (N = 42).[19]

Both software packages were developed in Unity. Augmented reality support for HoloLens 2 was enabled through the MRTK.
K-wire targets that were placed in accordance with the most typical locations for the implant in the elbow are also included in the virtual elbow model. For the sake of expediency, the candidate has the option to pick or unselect them. In addition, the candidate can view the virtualized position of the actual K-wire on the drill with the AR glasses in relation to the virtual elbow model.

Data logging system

Data logs are registered with two specifically designed software packages, namely the K-wire virtualization system and the AR K-wire placement test companion.
K-wire virtualization system is an Autodesk fusion 360 plugin written in python that virtualizes, via a set of coordinates relative to the phantom markers, the K-wire onto the virtual model measuring distances from key anatomical structures and discrepancy angles from K-wire target axis.
AR K-wire placement test companion is a tool written in python that measures parameters of the tests such as time, scintigraphy taken, confidence of the candidate, demographic Information and experience. The tool then stores all the data relative to candidate and K-wire placement on a local file database for later analysis.
The K-wire virtualization Fusion 360 plugin and companion are available at

- https://github.com/giacomo-riberi/kwire-virtualization-system
- https://github.com/giacomo-riberi/AR-kwire-placement-test-companion

Analysis

There are 3 database tables, PHASE, ECP, and PA, containing the relative data and statistics. The tables are connected through referential integrity constraints, where foreign key relationships link one table's primary key to another table's foreign key to reconstruct accurately all tests recorded.
Database structure based on unique ids and keys allows for easy data anonymization and secure storage to maintain participant confidentiality.
We divide analysis into statistical, positional, duration, Fluoroscopic image count and anatomical. Results and tables can be found in the “Results” section of this paper.

Supplementary Results

In this study, 19 applicants performed 150 Estimated Correct Positions (ECP) for a total of 193 Positioning Attempts (PA) across 50 phases. A total of 11·8 hours were spent for surgical simulation, together with 2542 fluoroscopic images.
A representative image that provides a clear visual understanding of the study’s measurements is presented in Fig. S3. It’s possible to observe that from phase 0 to 2, the bundle size is reduced.
34 (18·1%) PAs hit arteries, veins, or nerves with 43 contact instances.
31 (21·3%) ECPs hit arteries, veins, or nerves for a total of 43 contact instances.

Participant characteristics
8 medical master's students, 10 orthopedic residents, and 1 orthopedic surgeon participated to the study (supplementary table 1). The reported operation experience ($p < 0·01$) and age ($p < 0·01$) were linked with the participants' experience level, as expected. The participants' sex, familiarity with VR/AR technology, and proficiency in 3D software visualization did not conform to this pattern.
The only statistically significant parameter in supplementary table 2 is the insertion point error which is minimum on average in residents and students.
Supplementary table 3 clearly illustrates that across all three phases, X-ray only (Phase 0), initial AR use (Phase 1), and continued AR use (Phase 2), all four parameters displayed a statistically significant improvement.
The only variable that increases significantly in Supplementary table 4 is the angular error. This increase could be attributed to the progressively increasing distance between the target K-wire and the 3D camera.
In supplementary table 5, we observe that glasses have no influence on angular error, insertion point error, PA duration and PA fluoroscopic images.

Statistics
The number of ECPs reached after 1, 2 or 3 failed PA display a statistically significant drop for phase 1 and 2 where AR was used (fig.1A). Success rate increases by 23·1% between phase 0 and 1 and 24·6% between phase 0 and 2 with a $p<0·005$ proving a statistically significant improvement (fig.1B).

Duration
A clear and statistically significant time reduction is visible in fig. 2A, which is -123·47s between phase 0 and 1, and -142·96s between phase 0 and 2. ANOVA and Dunnett support the statistical validity of our measurements with a $p<0·005$.
It is crucial to remember that time to reach ECP holds greater significance compared to the time to finish a PA. This is because multiple PA might need to be performed to successfully place a designed target in a real-world scenario. Nevertheless, the graph illustrating the time to finish a PA with phase as predictor variable (not shown) reached similar conclusions to the above mentioned.

Fluoroscopy
The mean reduction of fluoroscopic images required to reach ECP (Fig.2B) is -28·87 (-84%) between phase 0 and 1, and -26·92 (-78%) between phase 0 and 2. This reduction is proven significant by the ANOVA and Dunnett tests performed on the database data with a $p<0·005$.

Position
The mean angle error drops in a statistically significant manner: compared to phase 0 (X-ray) by -2.80° (-27%) for phase 1 (AR first try) and -4·88° (-47%) for phase 2 (AR second try), indicating a substantial improvement in PA angular precision with AR (fig.3A). This reduction is proven

significant by the Dunnett test performed on the database data with a $p < 0{\cdot}005$ between phase 0 and 1 and a $p < 0.001$ between phase 0 and 2.
Also mean angle standard deviation is reduced significantly, indicating an increase in PA angle consistency. This reduction is proven significant, even after Bonferroni correction, by the Levene test performed on the database data with $p < 0{\cdot}005$ between phase 0 and 2 and $p < 0{\cdot}05$ between phase 1 and 2. No significant change was found between phase 0 and 1.
The mean PA entrance point distance from target is also significantly reduced (fig.3B): compared to phase 0 (X-ray) by -2·88mm (-32%) for phase 1 (AR first try) and -3·22mm (-36%) for phase 2 (AR second try), indicating a substantial improvement in PA precision. The standard deviation displays a progressive reduction, indicating a significant reduction in variability, and thus an increase in consistency and precision, of the PA skin insertion point distance from target skin insertion point (fig.3).

Anatomy
For target 1 on the phantom, PAs resulted in a non-significant change in the mean distance from the Ulnar nerve across phase 0, 1 and 2 (fig.4A). This is statistically confirmed as the ANOVA and Dunnett tests p-value indicate a non-significant mean change. This is expected as the mean is assumed to remain close to the target distance irrespective of the methodology employed, under the assumption of the absence of systematic errors. The data points indicating 0 from the ulnar nerve correspond to the PAs that struck the nerve. Assessment of the equality of variances displays a non-statistically significant change, which indicates that the placement precision remains constant across the three phases for this target.
The mean PA distance from Ulnar nerve from target 2 remains relatively stable across phases 0, 1 and 2 as the p values on the ANOVA and Dunnett tests indicate a non-significant mean change (fig 4B). This is expected as the mean is assumed to remain close to the target distance irrespective of the methodology employed, under the assumption of the absence of systematic errors. The standard deviation steadily decreases across the three phases, indicating a reduction in the variability of PA distance from Ulnar nerve leading to an increase of consistency. If we assess the equality of variances between phases, using the Levene test, we observe a statistically significant variance reduction, which correlates with an improvement in placement precision. Specifically, the variance difference between Phases 1 and 2 was 6.33 ($p < 0.05$, Bonferroni corrected), and the difference between Phases 0 and 2 was even greater, at 9.44 ($p < 0.01$, Bonferroni corrected).

Discussion

A representative image that can serve the reader to fully comprehend the study is rendered in fig. S3. The insertion displacement described by the Gaussianoid Conical Frustum~~,~~ which leads to increased iatrogenic risk when using AR for specific study cases, follows the analogy of two marksmen, one with a rifle and the other with a bow, shooting at an apple on someone's head. The former is more likely to hit the apple, but even a near miss might be fatal, whereas the latter is unlikely to strike the person as well as the fruit. If one merely considers the number of apple carriers killed and the average distance of arrow impacts from the target, the bow appears to be the better option. Yet, one must remember that the apple carrier is not alone in the field, and the

arrow has a much larger chance of injuring bystanders than the bullet. The same solution applies: the shooter's best choice is to use the rifle and have the target apple positioned where it would be safe shooting it with a bow. This effect is quite recurrent in everyday life, take a look at fig. S4 representing a rifle target: blades of grass very near the circle are at a very high risk of being hit by the rifle, which in the analogy represents AR, while grass at 10cm of distance can already be considered safe (high risk in a small radius range). We can easily understand how shooting at this target with a bow, representing x-ray, will result in less blades of grass being stuck for an overall much greater radius (medium risk in a much greater radius range).

References

[1] Wang Y, Tai BL, Yu H, Shih AJ. Silicone-based tissue-mimicking phantom for needle insertion simulation. Journal of Medical Devices. 2014;8(2):021001.
[2] Pogue BW, Patterson MS. Review of tissue simulating phantoms for optical spectroscopy, imaging and dosimetry. Journal of biomedical optics. 2006;11(4):041102-2.
[3] Savi M, Andrade MA, Potiens MP. Commercial filament testing for use in 3D printed phantoms. Radiation Physics and Chemistry. 2020;174:108906.
[4] El Mehtedi M, Buonadonna P, Carta M, El Mohtadi R, Marongiu G, Loi G, et al. Effects of milling parameters on roughness and burr formation in 3D-printed PLA components. Procedia Computer Science. 2023;217:1560-9.
[5] Cardoso SA, Suyambu J, Iqbal J, Jaimes DCC, Amin A, Sikto JT, et al. Exploring the role of simulation training in improving surgical skills among residents: a narrative review. Cureus. 2023;15(9).
[6] Aumann CA. A methodology for developing simulation models of complex systems. Ecological Modelling. 2007;202(3-4):385-96.
[7] Tan SSY, Sarker SK. Simulation in surgery: a review. Scottish medical journal. 2011;56(2):104-9.
[8] Ziv PRWA Stephen D Small. Patient safety and simulation-based medical education. Medical teacher. 2000;22(5):489-95.
[9] Cangelosi A, Riberi G, Salvi M, Molinari F, Titolo P, Agus M, et al. Mixed Reality for Orthopedic Elbow Surgery Training and Operating Room Applications: A Preliminary Analysis. Smart Tools and Applications in Graphics. 2023:74.

List of Figures

Fig. S1: building process of the phantom.
Fig. S2: testing setup: arrows describe information direction and type. Red: infrared positional data; Black: digital data; Blue: visual data; Green: AR data.
Fig. S3: K-wire bundles on the virtual phantom. The green K-wires represent the 3 target positions. All the 193 PAs are rendered across the 3 phases: phase 0 (left), 1 (center) and 2 (right).
Fig. S4: representing the same target seen from 2 different angles. The rifle has high precision but this paradoxically increases the strike risk just outside the radius of the target.

List of Videos

Vid. S5: POV of the full test procedure with one PA for each target.

List of Tables

Supplementary table 1: cross supplementary table for dependent career. Variables analyzed are gender, experience with virtual reality (VR), augmented reality (AR) and 3D editors, age, number of surgical operations and if the candidate wears glasses.

Supplementary table 2: cross supplementary table for dependent career. Variables analyzed are angular error, insertion point error, PA duration, PA fluoroscopic images taken.

Supplementary table 3: cross supplementary table for dependent phase. Variables analyzed are angular error, insertion point error, PA duration, PA fluoroscopic images taken.

Supplementary table 4: cross supplementary table for dependent target number. Variables analyzed are angular error, insertion point error, PA duration, PA fluoroscopic images taken.

Supplementary table 5: cross supplementary table for dependent glasses. Variables analyzed are angular error, insertion point error, PA duration, PA fluoroscopic images taken.

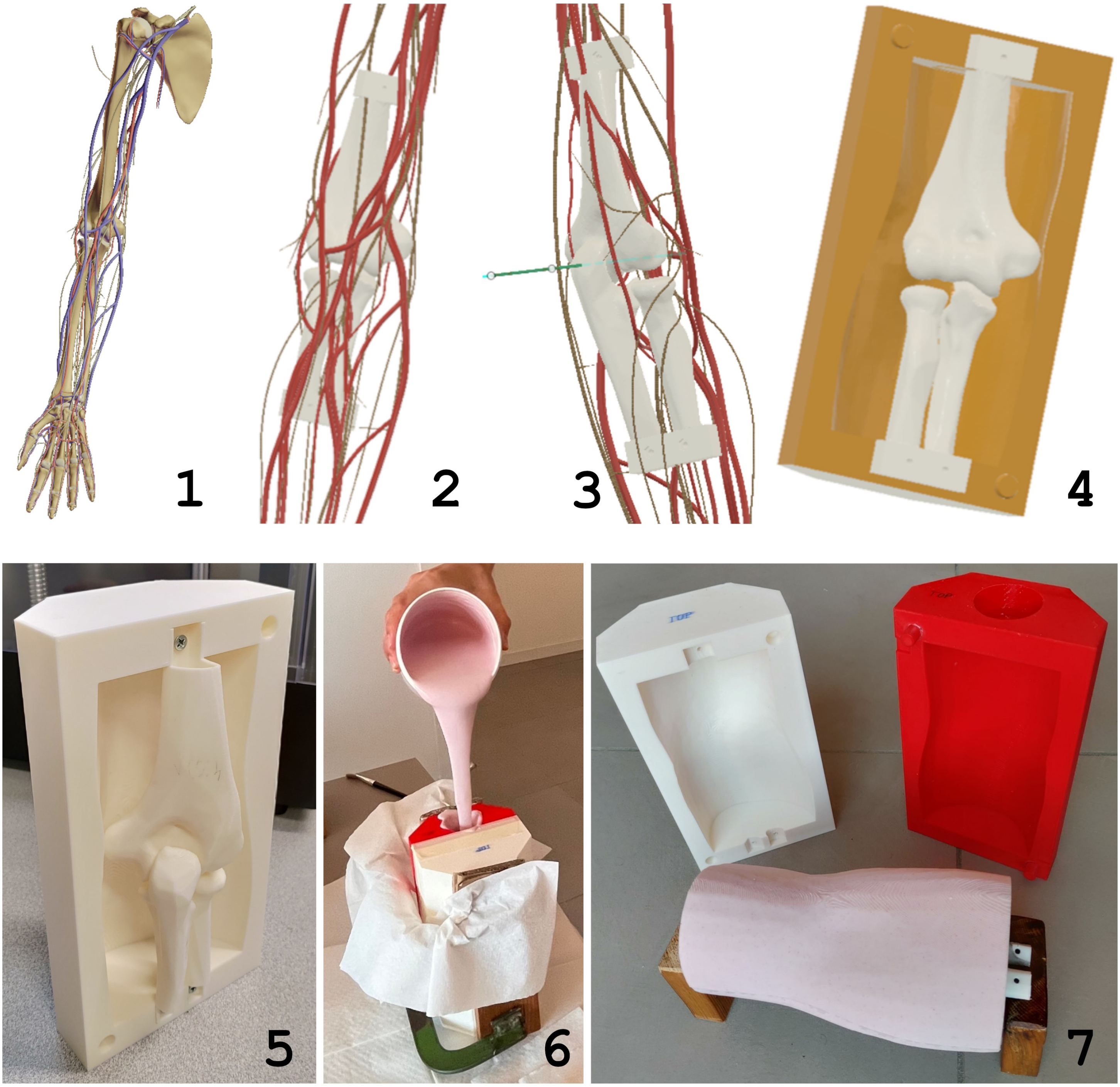

Fig. S1: building process of the phantom. 1) Z-anatomy model of right upper limb, including bones, vasculature and nerves. 2) Blender model of an extracted portion of the upper limb, from the third proximal to a third distal from the elbow. 3) Surgical planning from Fusion 360, where a k-wire placement is visible in green. 4) Fusion 360 model of the mold for the soft tissue casting. 5) 3D printed mold, with bones fixed in correct position. 6) Casting process of RTV silicon in a closed mold. 7) Final phantom extracted from the mold.

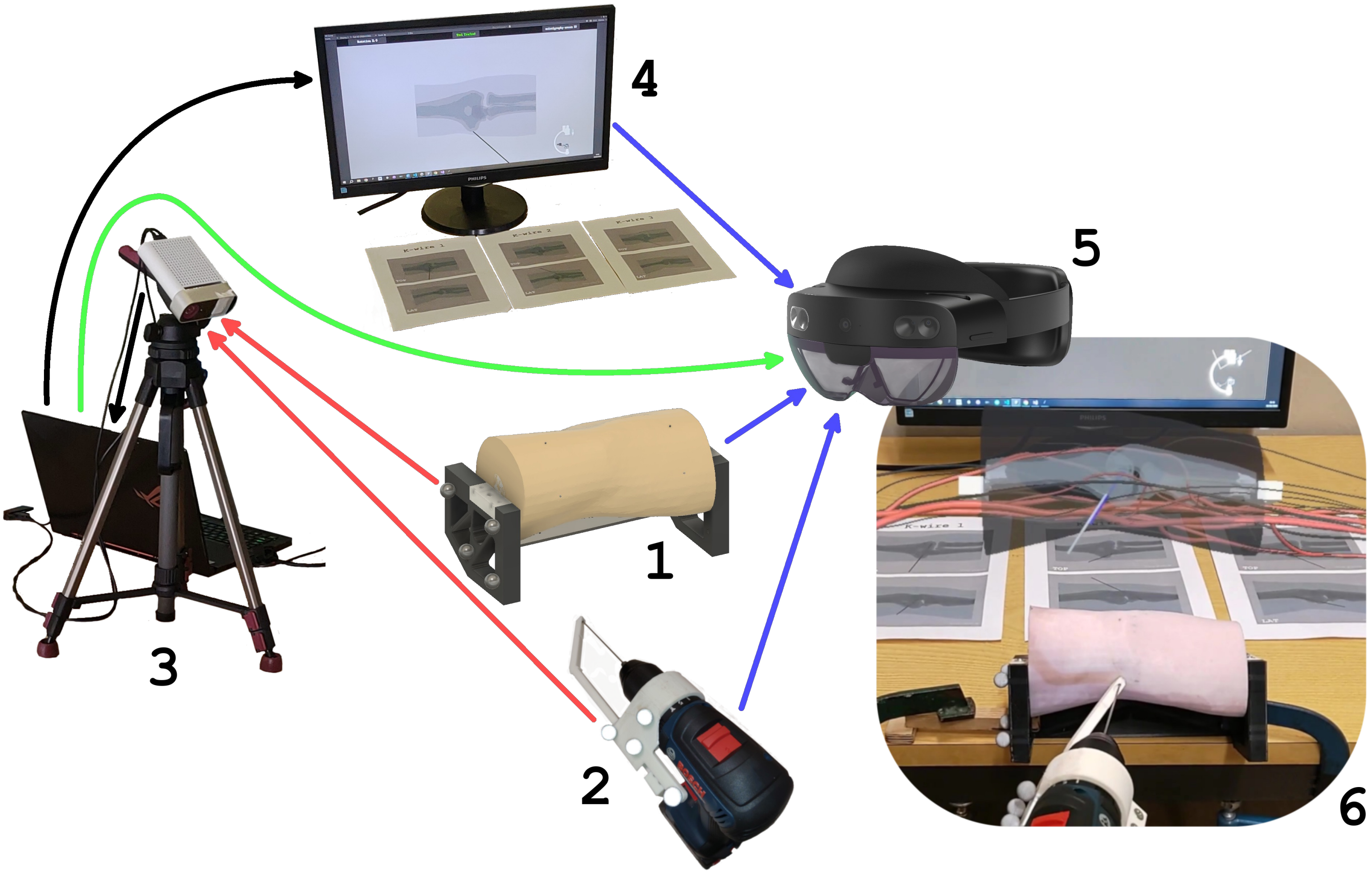

Fig. S2: testing setup. 1) Elbow phantom equipped with IR spheres. 2) Surgical drill equipped with tracking system and IR spheres. 3) Azure Kinect Camera for tracking. 4) Simulated X-ray system. 5) Microsoft Hololens HMD. 6) POV screenshot showing the virtual 3D model with the position of the k-wire tracked by the camera.
Arrows describe information direction and type. *Red*: infrared positional data; *Black*: digital data; *Blue*: visual data; Green: AR data.

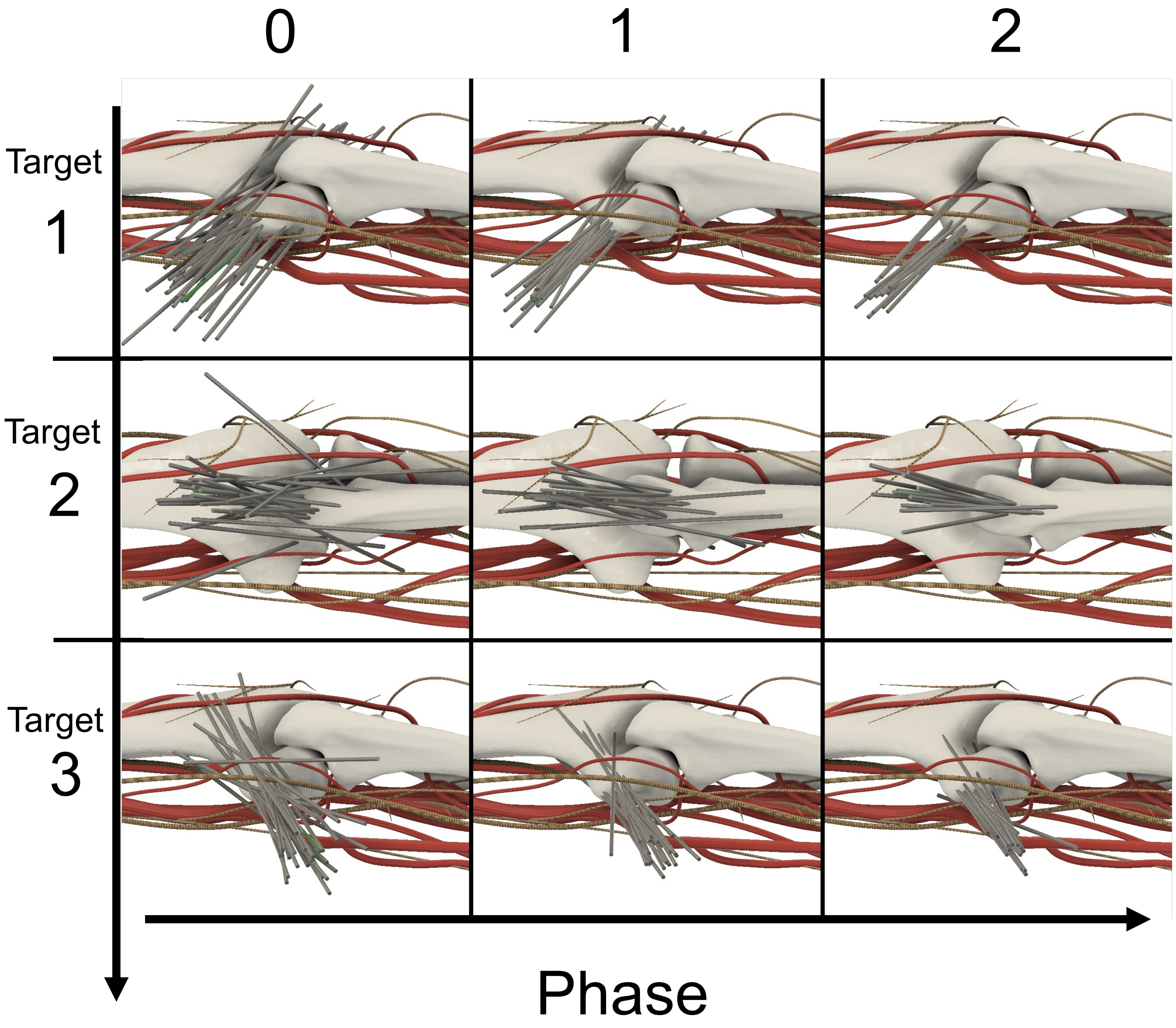

Fig. S3: **K-wire bundles on the virtual phantom.** Phase 0 (left), 1 (center) and 2 (right), all the 193 PAs are rendered. The green K-wires represent the 3 target positions (left).

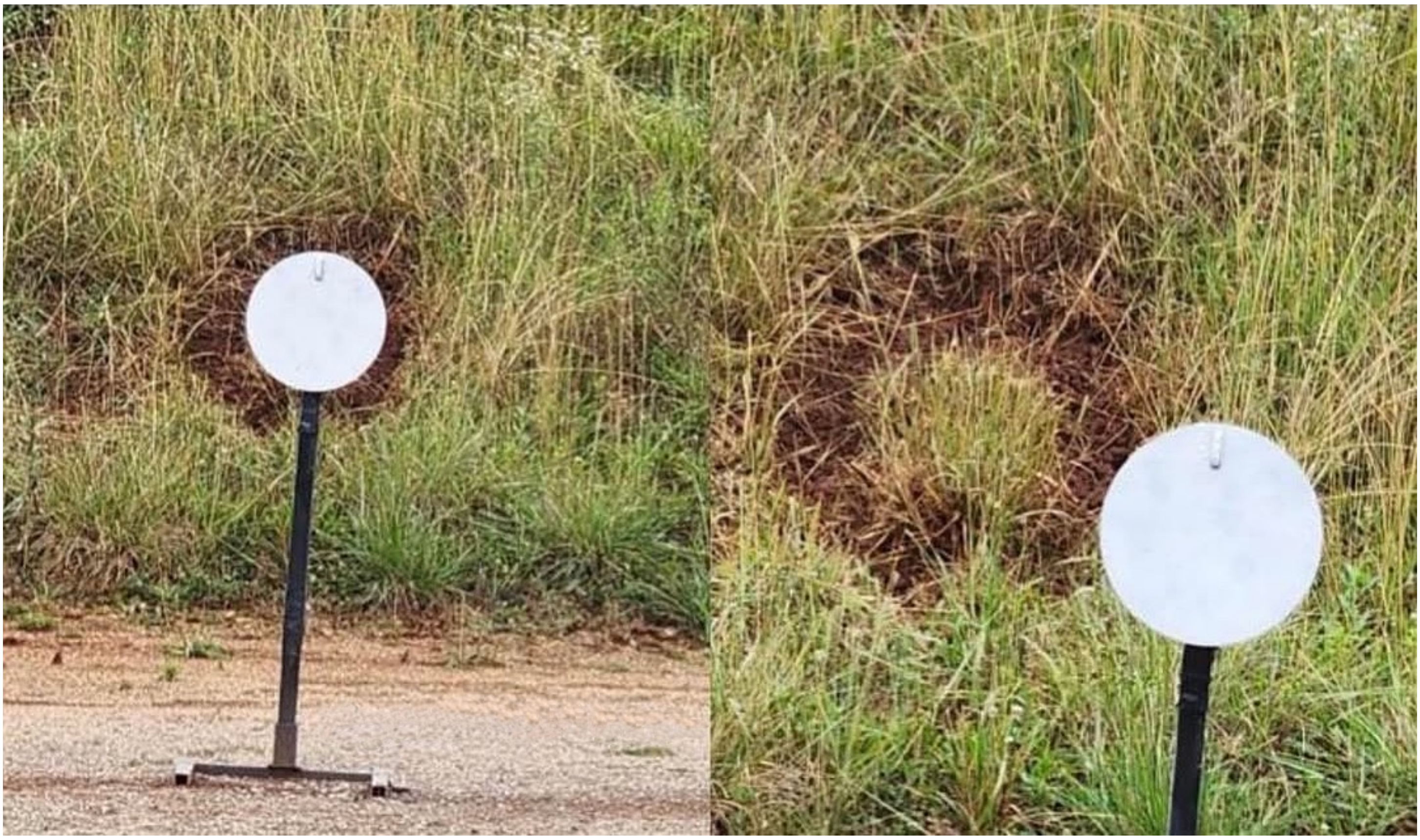

Fig. S4: **representing the same target seen from 2 different angles.** The rifle has high precision, but this paradoxically increases the strike risk just outside the radius of the target.

Cross Table for Dependent career

	N	student (N=8)	resident (N=10)	surgeon (N=1)	Test Statistic
gender : m	19	0.4 3/8	0.7 7/10	0.0 0/1	X^2_2=3.06, P=0.22[2]
experience VR	19				X^2_6=4.75, P=0.58[2]
0		0.5 4/8	0.5 5/10	1.0 1/1	
1		0.2 2/8	0.4 4/10	0.0 0/1	
2		0.0 0/8	0.1 1/10	0.0 0/1	
5		0.2 2/8	0.0 0/10	0.0 0/1	
experience AR	19				X^2_6=7.36, P=0.29[2]
0		0.8 6/8	0.6 6/10	0.0 0/1	
1		0.0 0/8	0.3 3/10	1.0 1/1	
2		0.1 1/8	0.1 1/10	0.0 0/1	
5		0.1 1/8	0.0 0/10	0.0 0/1	
experience 3D editor	19				X^2_{10}=9.42, P=0.49[2]
0		0.5 4/8	0.3 3/10	0.0 0/1	
1		0.1 1/8	0.3 3/10	0.0 0/1	
2		0.2 2/8	0.1 1/10	1.0 1/1	
3		0.0 0/8	0.2 2/10	0.0 0/1	
4		0.0 0/8	0.1 1/10	0.0 0/1	
5		0.1 1/8	0.0 0/10	0.0 0/1	
age	19	22.0 **23.5** 26.2	27.9 **29.0** 30.0	49.0 **49.0** 49.0	$F_{2,16}$=7.95, P<0.01[1]
experience operation count	19	0.0 **0.0** 0.0	5.0 **15.0** 30.0	500.0 **500.0** 500.0	$F_{2,16}$=44.83, P<0.01[1]
glasses : yes	19	0.8 6/8	0.6 6/10	1.0 1/1	X^2_2=0.95, P=0.62[2]

N is the number of non-missing value. [1]Kruskal-Wallis. [2]Pearson.

Supplementary Table 1

Cross Table for Dependent career

	N	student (N=91)	resident (N=96)	surgeon (N=6)	Test Statistic
Angular error	193	4.2 **7.8** 11.5	4.5 **6.7** 10.6	4.4 **7.6** 14.8	$F_{2,190}=0.61$, $P=0.54$[1]
Insertion point error	193	4.4 **7.5** 11.2	3.4 **5.3** 8.0	5.1 **6.3** 9.3	$F_{2,190}=6.74$, $P<0.01$[1]
PA duration	193	82.4 **124.2** 171.8	91.1 **151.0** 235.4	108.2 **142.0** 159.9	$F_{2,190}=2.52$, $P=0.08$[1]
PA radiation pictures	193	3.0 **6.0** 18.8	5.0 **7.5** 15.2	1.8 **6.0** 8.2	$F_{2,190}=1.11$, $P=0.33$[1]

N is the number of non-missing value. [1]Kruskal-Wallis.

Supplementary Table 2

Cross Table for Dependent phase

	N	0 (N=88)	1 (N=58)	2 (N=47)	Test Statistic
Angular error	193	5.6 **9.7** 12.6	3.5 **6.6** 9.5	3.8 **4.7** 7.4	$F_{2,190}=16.09$, $P<0.01$[1]
Insertion point error	193	5.4 **8.1** 11.3	3.5 **4.8** 8.4	3.4 **5.5** 7.5	$F_{2,190}=9.59$, $P<0.01$[1]
PA duration	193	112.1 **158.2** 228.8	87.3 **118.6** 187.7	70.8 **109.5** 164.4	$F_{2,190}=7.88$, $P<0.01$[1]
PA radiation pictures	193	7.0 **14.0** 27.2	1.0 **4.0** 6.0	2.2 **5.0** 9.0	$F_{2,190}=52.00$, $P<0.01$[1]

N is the number of non-missing value. [1]Kruskal-Wallis.

Supplementary Table 3

Cross Table for Dependent Target number

	N	1 (N=65)	2 (N=66)	3 (N=62)	Test Statistic
Angular error	193	3.8 **5.3** 9.2	4.7 **7.8** 11.5	4.7 **8.2** 11.7	$F_{2,190}$=4.16, P=0.02[1]
Insertion point error	193	4.1 **6.2** 9.5	4.1 **6.6** 10.9	3.4 **5.5** 8.1	$F_{2,190}$=2.26, P=0.11[1]
PA duration	193	80.4 **121.7** 173.8	100.9 **137.1** 193.6	83.4 **142.2** 205.7	$F_{2,190}$=1.87, P=0.16[1]
PA radiation pictures	193	3.7 **6.0** 13.3	3.9 **6.0** 17.2	3.9 **8.0** 16.0	$F_{2,190}$=0.35, P=0.70[1]

N is the number of non-missing value. [1]Kruskal-Wallis.

Supplementary Table 4

Cross Table for Dependent glasses

	N	no (N=59)	yes (N=134)	Test Statistic
Angular error	193	4.7 **8.0** 11.7	4.2 **6.9** 10.7	$F_{1,191}$=1.11, P=0.29[3]
Insertion point error	193	3.6 **6.1** 9.0	3.9 **6.2** 9.4	$F_{1,191}$=0.03, P=0.87[3]
PA duration	193	90.7 **131.7** 185.4	83.4 **131.3** 197.1	$F_{1,191}$=0.04, P=0.85[3]
PA radiation pictures	193	4.2 **7.0** 12.7	3.0 **7.0** 17.0	$F_{1,191}$=0.02, P=0.88[3]

N is the number of non-missing value. [3]Wilcoxon.

Supplementary Table 5
